# FSH levels and testicular volumes are associated with the severity of testicular histopathology in men with non-obstructive azoospermia

**DOI:** 10.1007/s10815-021-02313-y

**Published:** 2021-09-17

**Authors:** Parviz K. Kavoussi, Kayla Hudson, G. Luke Machen, Maya Barsky, Dan I. Lebovic, Shahryar K. Kavoussi

**Affiliations:** Austin Fertility & Reproductive Medicine/Westlake IVF, 300 Beardsley Lane, Building B, Suite 200, Austin, TX 78746 USA

**Keywords:** Non obstructive azoospermia, Histopathology, Follicle stimulating hormone, Testicular volume

## Abstract

**Purpose:**

The purpose of this study is to assess a potential association between FSH levels and testicular volumes with the severity of testicular histopathology on testicular biopsy in men with non-obstructive azoospermia (NOA) undergoing microdissection testicular sperm extraction (microTESE).

**Methods:**

A retrospective chart review was performed from the electronic health records of men who underwent microTESE with NOA.

**Results:**

Eighty-six men with NOA underwent microTESE with concomitant testicular biopsy for permanent section to assess the testicular cellular architecture. The histopathological patterns were categorized by severity indicating the odds of sperm retrieval into 2 categories. The unfavorable category included Sertoli cell only pattern and early maturation arrest (*n* = 50) and the favorable category included late maturation arrest and hypospermatogenesis patterns (*n* = 36). In the men with unfavorable histopathologic patterns, the mean FSH level was 22.9 ± 16.6 IU/L, and the mean testicular volume was 10.4 ± 6.0 cc. This was in comparison to men with favorable histopathologic patterns revealing a mean FSH level of FSH 13.3 ± 12.0 with a mean testicular volume of 13.3 ± 5.9 cc. There was a statistically significant higher FSH level in men with unfavorable histopathology than favorable (*p* = 0.004) as well as a significant smaller mean testicular volume in men with unfavorable histopathology (*p* = 0.029).

**Conclusions:**

Higher serum FSH levels and smaller testicular volumes are associated with more severe testicular histopathological patterns in men with NOA.

## Introduction

One percent of men in the general population and 5–10% of men undergoing evaluation for infertility in the USA are azoospermic [1, 2]. Microdissection testicular sperm extraction (microTESE) offers the largest number of sperm cells retrievable from men with non-obstructive azoospermia (NOA) with primary testicular dysfunction, for use with in vitro fertilization/intracytoplasmic sperm injection (IVF/ICSI) [3–6]. The combination of an elevated serum follicle stimulating hormone (FSH) level greater than 7.6 IU/L and smaller testicular volumes with a long axis of 4.6 cm or less predicts the etiology of azoospermia being due to spermatogenic dysfunction [7]. This has led to testis biopsy rarely being indicated in the diagnostic evaluation to differentiate between obstructive azoospermia and NOA. However, it is common practice for a testicular biopsy to be obtained for permanent section at the time of microTESE to help define the testicular histopathology and the severity of the testicular dysfunction.

The 4 testicular histopathological tissue diagnoses in men with NOA include hypospermatogenesis, early maturation arrest, late maturation arrest, and Sertoli cell only pattern. The least severe histopathology seen in NOA testicular biopsy samples is hypospermatogenesis, which reveals general depopulation of the seminiferous epithelium, but a variety of germ cells in all stages of spermatogenesis are visualized, with the seminiferous epithelium appearing atrophic with diminished luminal caliber. Maturation arrest describes patterns with complete interruption of spermatogenesis across all seminiferous tubules. Late maturation arrest refers to varying stages of spermatogenesis without completely formed sperm during or after meiotic division, such as spermatids but no spermatozoa. Alternatively, early maturation arrest refers to varying stages of spermatogenesis without completely formed sperm prior to meiotic division at the spermatogonia or the spermatocyte stage. The most severe histopathology is Sertoli cell only pattern, characterized by entirely absent germ cells with only Sertoli cells visible in the germinal epithelium by low-power light microscopy. It has been demonstrated that men with more severe histopathological architecture have lower sperm retrieval rates [8–13].

Although a number of studies have examined multiple clinical factors as predictors of microTESE sperm retrieval rates and few have evaluated predictors of fertilization, pregnancy, and live birth rates, this study aims to assess whether there is an association between FSH levels and testicular volumes with the severity of the testicular histopathology in men with NOA [9, 10, 12, 14–25].

## Materials and methods

Between January 2012 and April 2021, 86 men who presented for infertility evaluations at a couple’s fertility center were diagnosed with NOA by a single reproductive urologist (PKK). The diagnostic evaluation included a thorough history and physical examination, including testicular volume measurements by Prader orchidometer, 2 semen analyses with centrifugation revealing azoospermia, serum hormone testing, and genetic testing including a karyotype and a Y chromosome microdeletion assay. The diagnosis of NOA was made by the findings of atrophic testicular volumes on physical examination, normal semen volumes, and elevated FSH levels, consistent with primary testicular dysfunction. Men with any sperm isolated in the centrifuged semen sample consistent with cryptozoospermia were excluded from the data. The data included were from consecutive NOA patients who opted for microTESE/IVF/ICSI. Patients who did not choose to move forward with these treatments due to cost, religious reasons, or simply not interested in that level of treatment opted for other routes of building their family such as therapeutic donor sperm insemination or adoption and were excluded from the data. The men who elected to proceed underwent microTESE by a single reproductive urologist, at which time a testicular biopsy was performed and prepared for pathology for permanent section. Histopathology was interpreted by a pathologist at the operating hospital as well as confirmed by a second pathologist at Mayo clinic. Our technique for isolating sperm from the microTESE specimens includes having 2 embryologists present in the operating room during microTESE processing samples and searching for sperm intraoperatively. The specimens were then taken back to the IVF laboratory and further processed and assessed for a number of hours and left to culture overnight with further processing and assessment the following day.

After St. David’s Healthcare Institutional Review Board exemption (1,759,181–1) for electronic health record review and maintenance of data in a deidentified manner was obtained, a retrospective chart review was performed on records of these men. Statistical analysis was performed with the Student *t* test, with a *p* value of < 0.05 considered statistically significant. Results were expressed as means ± standard deviations.

## Results

Of the total of 86 men who underwent microTESE for NOA, 61/86 (71%) had sperm retrieved. The mean age of the men was 35.6 ± 7.3 years of age, and they had a mean testicular volume of 11.6 ± 7.3 CC, with a mean FSH level of 18.9 ± 15.5 IU/L. The permanent section pathology results from these men revealed that 29/86 (34%) had testicular architecture consistent with hypospermatogenesis, 7/86 (8%) with late maturation arrest, 1/86 (1%) with early maturation arrest, and 49/86 (57%) with Sertoli cell only pattern. The results were then categorized by favorable testicular histopathology for sperm retrieval (*n* = 36) including hypospermatogenesis and late maturation arrest patterns which had a combined 91.7% sperm retrieval rate, versus unfavorable testicular histopathology patterns (*n* = 50) including early maturation arrest and Sertoli cell only patterns which had a combined 56% sperm retrieval rate. FSH levels and testicular volumes were compared between the favorable histopathology and the unfavorable histopathology groups. Men with unfavorable histopathology had statistically significant higher levels of FSH and smaller testicular volumes (Table 1). Overall, 68/86 (79.1%) of the men had elevated FSH levels, and 68/86 (79.1%) had testicular atrophy. Of those with unfavorable testicular histopathology, 47/50 (94%) had elevated FSH levels, and 42/50 (84%) had testicular atrophy, while 21/36 (58.3%) had elevated FSH levels, and 26/36 (72%) had testicular atrophy in the favorable pathology group.

**Table 1:** Comparison of testicular volumes in cubic centimeters and FSH levels in IU/L for men with NOA with testicular histopathology that was favorable (hypospermatogenesis and late maturation arrest) versus unfavorable (early maturation arrest and Sertoli cell only pattern). Results expressed as means ± standard deviations. Statistical analysis for *p* values performed by Student *t* test

	Testis volume	FSH
Favorable pathology (*n* = 36)	13.3 ± 5.9	13.3 ± 12.0
Unfavorable pathology (*n* = 50)	10.4 ± 6.0	22.9 ± 16.6
*P* value	0.029	0.004

The mean serum total testosterone level in the men with unfavorable histopathology was 300 ng/dL ± 173, and the mean in the favorable group was 310 ng/dL ± 148. There was not a statistically significant difference between testosterone levels in the 2 groups, *p* value 0.78.

## Discussion

NOA is considered the most challenging scenario in couples fertility care and requires a high level of treatment for both partners. The advent of microTESE significantly improved the odds for these couples to have an opportunity to conceive with autologous gametes. The odds of sperm retrieval with microTESE have been associated with severity of the testicular histopathological architectural pattern in the parenchyma of the testis [8–13]. The outcomes of the men in this study were consistent with a significantly higher sperm retrieval rate of 91.7% in men with favorable histopathological patterns as opposed to 56% sperm retrieval rates in the men with unfavorable histopathological patterns.

The differentiation between azoospermia due to obstruction and NOA due to spermatogenic dysfunction previously required a diagnostic testicular biopsy to assess the level of spermatogenesis prior to offering definitive therapeutic options. More recently, the clinical findings of an elevated serum follicle stimulating hormone (FSH) higher than 7.6 IU/L and smaller volume testicles with a long axis of 4.6 cm or less has been established to predict the etiology of azoospermia to be due to spermatogenic dysfunction, or NOA [7]. The objective of this study was to assess whether the clinical findings of testicular volumes and FSH levels correlate with the severity of the histopathology identified in the testes of men with NOA undergoing microTESE.

The findings of more severe histopathological patterns of early maturation arrest and Sertoli cell only not only significantly correlated with higher FSH levels and smaller testicular volumes, but revealed great significance statistically with *p* values of 0.004 and 0.029, respectively. Therefore, FSH and testicular volumes may not only assist in differentiating between obstructive azoospermia and NOA but may also indicate the severity of and testicular cellular architecture in men with NOA.

A limitation to this study is the sample size; however, appropriate statistical analysis was performed. Another limitation is the understanding that the testicular histopathology may be heterogeneous throughout the parenchyma of the testis, although it has been well established that a testicular biopsy such as those performed in the men in this study is the standard for assessment of testicular histopathology [7–9, 11–13].

## Conclusions

Higher serum FSH levels and smaller testicular volumes are associated with more severe testicular histopathology in men with NOA.

## References

[1] Jarow JP, Espeland MA, Lipshultz LI (1989). Evaluation of the azoospermic patient. J Urol.

[2] Mazzilli F, Rossi T, Delfino M, Sarandrea N, Dondero F (2000). Azoospermia: incidence, and biochemical evaluation of seminal plasma by the differential pH method. Panminerva Med.

[3] Schlegel PN, Palermo GD, Goldstein M (1997). Testicular sperm extraction with intracytoplasmic sperm injection for nonobstructive azoospermia. Urology.

[4] Tsujimura A, Matsumiya K, Miyagawa Y (2002). Conventional multiple or microdissection testicular sperm extraction: a comparative study. Hum Reprod.

[5] Ramasamy R, Padilla WO, Osterberg EC (2013). A comparison of models for predicting sperm retrieval before microdissection testicular sperm extraction in men with nonobstructive azoospermia. J Urol.

[6] Ramasamy R, Reifsnyder JE, Husseini J, Eid PA, Bryson C, Schlegel PN (2013). Localization of sperm during microdissection testicular sperm extraction in men with nonobstructive azoospermia. J Urol.

[7] Schoor RA, Elhanbly S, Niederberger CS, Ross LS (2002). The role of testicular biopsy in the modern management of male infertility. J Urol.

[8] Aydin T, Sofikerim M, Yucel B, Karadag M, Tokat F (2015). Effects of testicular histopathology on sperm retrieval rates and ICSI results in non-obstructive azoospermia. J Obstet Gynaecol.

[9] Li H, Chen LP, Yang J (2018). Predictive value of FSH, testicular volume, and histopathological findings for the sperm retrieval rate of microdissection TESE in nonobstructive azoospermia: a meta-analysis. Asian J Androl.

[10] Berookhim BM, Palermo GD, Zaninovic N, Rosenwaks Z, Schlegel PN (2014). Microdissection testicular sperm extraction in men with Sertoli cell-only testicular histology. Fertil Steril.

[11] Abdel Raheem A, Garaffa G, Rushwan N (2013). Testicular histopathology as a predictor of a positive sperm retrieval in men with non-obstructive azoospermia. BJU Int.

[12] Kavoussi PK, West BT, Chen SH (2020). A comprehensive assessment of predictors of fertility outcomes in men with non-obstructive azoospermia undergoing microdissection testicular sperm extraction. Reprod Biol Endocrinol.

[13] Weedin JW, Bennett RC, Fenig DM, Lamb DJ, Lipshultz LI (2011). Early versus late maturation arrest: reproductive outcomes of testicular failure. J Urol.

[14] Tsujimura A, Matsumiya K, Miyagawa Y (2004). Prediction of successful outcome of microdissection testicular sperm extraction in men with idiopathic nonobstructive azoospermia. J Urol.

[15] Ramasamy R, Lin K, Gosden LV, Rosenwaks Z, Palermo GD, Schlegel PN (2009). High serum FSH levels in men with nonobstructive azoospermia does not affect success of microdissection testicular sperm extraction. Fertil Steril.

[16] Bernie AM, Ramasamy R, Schlegel PN (2013). Predictive factors of successful microdissection testicular sperm extraction. Basic Clin Androl.

[17] Bryson CF, Ramasamy R, Sheehan M, Palermo GD, Rosenwaks Z, Schlegel PN (2014). Severe testicular atrophy does not affect the success of microdissection testicular sperm extraction. J Urol.

[18] Ramasamy R, Trivedi NN, Reifsnyder JE, Palermo GD, Rosenwaks Z, Schlegel PN (2014). Age does not adversely affect sperm retrieval in men undergoing microdissection testicular sperm extraction. Fertil Steril.

[19] Modarresi T, Hosseinifar H, Daliri Hampa A (2015). Predictive factors of successful microdissection testicular sperm extraction in patients with presumed sertoli cell-only syndrome. Int J Fertil Steril.

[20] Cetinkaya M, Onem K, Zorba OU, Ozkara H, Alici B (2015). Evaluation of microdissection testicular sperm extraction results in patients with non-obstructive azoospermia: independent predictive factors and best cutoff values for sperm retrieval. Urol J.

[21] Xu T, Peng L, Lin X, Li J, Xu W. Predictors for successful sperm retrieval of salvage microdissection testicular sperm extraction (TESE) following failed TESE in nonobstructive azoospermia patients. Andrologia*.* 2017;49(4). 10.1111/and.12642. 10.1111/and.12642 27444399

[22] Althakafi SA, Mustafa OM, Seyam RM, Al-Hathal N, Kattan S (2017). Serum testosterone levels and other determinants of sperm retrieval in microdissection testicular sperm extraction. Transl Androl Urol.

[23] Alfano M, Ventimiglia E, Locatelli I (2017). Anti-Mullerian hormone-to-testosterone ratio is predictive of positive sperm retrieval in men with idiopathic non-obstructive azoospermia. Sci Rep.

[24] Yucel C, Budak S, Keskin MZ, Kisa E, Kozacioglu Z (2018). Predictive factors of successful salvage microdissection testicular sperm extraction (mTESE) after failed mTESE in patients with non-obstructive azoospermia: long-term experience at a single institute. Arch Ital Urol Androl.

[25] Kizilay F, Semerci B, Simsir A, Kalemci S, Altay B (2019). Analysis of factors affecting repeat microdissection testicular sperm extraction outcomes in infertile men. Turk J Urol.

